# Mental Health Promotion in School: Schoolchildren's and Families' Viewpoint

**DOI:** 10.1155/2014/395286

**Published:** 2014-11-19

**Authors:** Kristiina Puolakka, Anne Konu, Irma Kiikkala, Eija Paavilainen

**Affiliations:** ^1^Department of Psychiatry, Satakunta Hospital District, Talvitie 7, 29200 Harjavalta, Finland; ^2^University of Tampere, School of Health Sciences, 33014 Tampere, Finland; ^3^University of Eastern Finland, Käsityöläisentie 18 S 67, 00750 Helsinki, Finland; ^4^University of Tampere, School of Health Sciences, Nursing Science/Etelä-Pohjanmaa Hospital District, 33014 Tampere, Finland

## Abstract

While developing mental health work in schools, it is very important to consider the viewpoint of pupils. Parents can also give remarkable information on their children's viewpoint. The purpose of this study was to produce a description of the concepts used by schoolchildren aged 12–16 years and their families associated with promoting mental health in schools. The research material comprised interviews with schoolchildren and mothers, and verbal answers from the school well-being profile survey (*n* = 426). The analysis was conducted by applying the grounded theory method as introduced by Strauss. The study was conducted in a Finnish comprehensive school.

## 1. Introduction

Schoolchildren's biggest health problems are related to mental health. According to various estimates, the prevalence of mental health problems among adolescents varies between 10 and 30%. The most common disorders are depression, substance abuse, disturbance of attention, and eating disorders. Emotional problems are more common among girls. Boys' problems manifest in outwardly disruptive behaviour [1–8].

Mental health is fundamental to the good health and quality of life. It is defined by WHO as a state of well-being in which every individual realizes his or her own potential, can cope with the normal stresses of life, can work productively and fruitfully, and is able to make a contribution to her or his community [9]. Positive mental health is seen as a resource which is essential to general well-being. The promotion of mental health is a more extensive concept than merely preventing mental health problems. Mental health promotion strives to find and enhance factors and processes that protect mental health and reduce factors harmful to it. Mental health promotion can improve people's survival skills and ability to feel empathy and, consequently, protect their mental health and improve their ability to support other members of their community with mental health problems. The promotion of mental health not only consists of the support of the mental health of individuals and provision of mental health services but also includes activities at the community and society levels [10–14]. Actions to promote mental health include national mental health programmes, laws and policies, development of mentally healthy communities and physical environments and providing of opportunities for leisure activities [14]. As mental health is an integral part of health, mental health promotion is an integral part of overall health promotion [11].

An environment that promotes or hinders schoolchildren's mental health involves the person's entire mental, physical, and social environment, especially the school, home, and friends [15]. There is evidence of how mental health promotion in schools has positive effects on the age group. Taking into account the whole of the school community especially, environment and the families of the pupils as well as focusing on positive mental health and adequate lengths of the treatments are factors which contribute to the success of mental health promotion. The effects are personal, social, and economical [16–20]. Preventing mental health problems and doing mental health promotion have an effect in one's success in school and well-being in life in general. The mental health and behavioural problems of children and adolescents can often be seen as difficulties in later phases of their lives [21–25].

Barry and Jenkins [10] made an overview of the evidence from systematic reviews and several mental health promoting programmes. They named characteristics of successful school-based interventions. These are high-quality implementation, evaluation and sustainability and such things as adopting a whole-school and a social competence approach, performing interventions over multiple years and having a strong theoretical base. Used theoretical foundations are mostly psychology theories such like child development theory or cognitive behavioural therapy but in many programs there is no theory basis at all [10, 26].

Cooperation and the participation of all community members in mental health promotion have been found to be significant factors for the success of planned mental health programs [4, 10, 27, 28]. Self-expression and self-ruling are key factors for well-being; therefore, just being heard affects mental health and well-being [27–29]. WHO European Ministerial Conference also focuses in its Mental Health Declaration fostering awareness of importance of mental well-being (priority 1) and recognizing knowledge and experiences of service users and carers (family member, friend, or another informal caregiver) as an important basis on planning and developing mental health services (priority 5) [30]. Schoolchildren are the main target of mental health promotion, which makes them key elements in the discussion on promoting mental health at school. The family plays a key role. It is the primary, most important, and most influential system to which the child belongs [31]. Schoolchildren and their families represent service users in this study material.

Despite the large number of projects and recommendations on the promotion of schoolchildren's mental health, the literature does not offer a comprehensive theoretical description of what mental health work with schoolchildren is as a whole [10, 26]. Having a theoretical description of mental health promotion work helps in the development of facilities [10, 26, 28]. A theory can be constructed by combining and comparing the viewpoints of different parties—the employees, the schoolchildren, and their families—as well as previous knowledge of the subject [32].

This study is a part of a more extensive research to produce a theory of mental health promotion in the upper level of comprehensive school. In these schools, pupils' ages are from 12 to 16. This study aims at describing the concepts referring to the promotion of mental health from the schoolchildren's and their families' perspective and, consequently, facilitating the development of the practices of mental health promotion. In this study, families' perspective was studied by interviewing mothers of schoolchildren.

In this study, it was intended to especially highlight the perspective of the schoolchildren and their families. Therefore, the analysis was left on the level of axial coding to produce and describe the concepts. After that, it is easier to develop practices and support their self-ruling ability [32]. Schoolchildren are the main targets of mental health promotion, which makes them key elements in the discussion on promoting mental health at school. Self-expression and self-rule are key factors for well-being; therefore, just being heard affects mental health and well-being [27–29].

The purpose of this study was to find the key aspects and requirements for promoting mental health in the way the pupils and their families see them. The research question in this study was the following. What is mental health promotion from the schoolchildren's and families' perspective?

## 2. Materials and Methods

### 2.1. Research Environment

This study was conducted in a Finnish upper-level comprehensive school with 446 pupils in 2005–2007. This is the only upper-level comprehensive school in the quite small city where the school is located. In Finland, children start the 9-year comprehensive school at the age of 7. The first 6 grades comprise the primary school. These schools are usually smaller and closer to the pupils' homes. The last three grades of comprehensive school—attended from age of 12 to age of 16—are usually, and also in this case, located in a bigger school further away from the homes [33]. The school offers an excellent environment to reach the whole age group because of the overarching school system. In Finland, almost every child (99.7%) completes the nine-year-long basic education [34].

### 2.2. Study Material

This study used two types of research material. The first type consisted of material gathered from the interviews with school pupils and their parents. The parents who agreed to be interviewed were all mothers, but they were asked to speak from the viewpoint of the whole family. The second type consisted of the pupils' written replies to the well-being survey conducted by their school. The school well-being profile is a tool that is used to measure well-being in school communities. It consists of 80 structured questions and two open questions. The open questions were as follows: “what is the best feature in your school?” and “what things in your school should especially be improved?” [35]. Because positive mental health is an intregral part of general well-being, it was assumed that statements regarding mental health could be found in this study's survey material [12]. In this study only the answers to the open questions were used as a material. The responses to these two questions were incorporated into the other data sets and the statements drawn from them were analysed together with the rest of the data. The researcher also wrote memos, which were used to support the analysis [32]. The research was conducted in accordance with universal ethical principles [36].

The collection of material was carried out in parallel with the analysis. A sufficient amount of data was considered having been collected when the analysis no longer resulted in statements that did not fit into the categories [32]. The interviews with the schoolchildren and mothers produced 54 pages of text and the verbal answers to the school well-being profile produced 436 statements that were also added to the research material. A total of 1523 statements were written up as a result of the interviews and the survey questions.

The themes of the interviews were as follows:

- *the conceptions of the interviewees of mental health work in general and in this school;*
- *school as a working environment;*
- *particular concerns, such as bullying and absences, and actions to prevent them;*
- *getting help in mental health problem situations; cooperation between the home of the pupil and the school;*
- *cooperation between primary health care, social services, and special health care;*
- *the most crucial development needs in mental health promotion of schoolchildren.*

Two boys, three girls, and four mothers participated in the interviews. The interviews were conducted by the researcher and they were carried out in the school during the school day.

The principal was aware of the aims and methods of the study and of the fact that there was a need for interviewees who have things to say through their own experience and who represent the schoolchildren comprehensively. The interviewees were thus chosen with the choosing criteria of qualitative research in mind [32]. She approached a group of parents and these four were chosen to be the respondents due to their willingness to participate. Principal also sent permission forms and information about the study to the parents. Finally, the information was signed by the researcher. The principal did not participate in the interview situations. The criteria for selecting the pupils to be interviewed were that the pupils represented different grades and were of both sexes.

The relatively open interview themes helped to see the world from the perspective of participants and to develop deeper understanding of their viewpoint [32]. In the interview material of the parents, saturation was achieved easily, because the material was very rich. For the schoolchildren, it seemed to be easier to produce statements anonymously in the survey than it was in the interview. With the survey material, it was possible to reach many schoolchildren and get their opinions on their school. The open questions in the survey also provided a way to find aspects the schoolchildren themselves found important. In an interview situation, the answers may sometimes be influenced by the interview themes and questions. The survey was executed during a school day. The school nurse handed out and collected the questionnaires. The survey was directed to all of the pupils in the school. The school well-being profile survey was completed by 423 out of 426 pupils present in school on the day of the survey.

### 2.3. The Method: Grounded Theory

The grounded theory method was applied in the data collection and analysis. Grounded theory is a systematic method for that purpose. It aims at creating a substantive theory, including concepts and assumptions on the relationships among them [32, 37, 38]. Grounded theory was selected because it is analysing method that takes account of people and their experiences. In grounded theory, the researcher aims to come close to the actor's perspective and tries to capture his or her viewpoint [39]. This perspective of schoolchildren and families is needed to understand the mental health promotion in school. To understand experience, it must be located within the larger events in a social, political, cultural, ethnic and gender related, informational, and technological framework. These are essential aspects of grounded theory analysis. Grounded theory elevates the everyday empirical knowledge to a conceptual level. Concepts provide ways of talking about and arriving at shared understanding among professionals. Developing knowledge helps to develop practice [32, 40].

The theoretical background of the grounded theory method lies in symbolic interactionism and pragmatism. Symbolic interactionism brings attention to human behaviour and interaction by focusing on the significance of events and things in people's everyday lives. It is useful for conceptualising complex situations, understanding unsolved social problems, and understanding the impact of new ideologies. Pragmatism assumes that knowledge is created through action and interaction [32].

The objective of grounded theory is to generate basic models as explanations of social life in general. The data are collected from everyday life [32, 37, 41]. There are two main tendencies regarding the inductiveness of the analysis. One, the purely inductive method represented by Glaser, develops theory purely on the basis of the material. The other, favoured by Strauss and applied in this study, inspects the existing theoretical knowledge and combines it with the data gained through research [32, 37, 39]. Because of the relatively large number of articles about some aspects of mental health promotion at school, it is reasonable to examine and use previous articles about the promotion of schoolchildren's mental health while producing a comprehensive theoretical description. This is why the Strauss method was selected for this study [32, 37, 39]. The schoolchildren's and families' perspective that comes from the material of this study can be compared to the existing knowledge and viewpoints [37, 39].

### 2.4. Ethical Aspects Related to the Research

The research was conducted in accordance with universal ethical principles. Some points should be taken into account when under-age respondents are used in a study. Above all, it is important to acquire all appropriate permissions [36]. In Finland, respondents aged 12 to 16 are considered old enough to participate in some cases even without permission from their parents. Such cases are situations where sensitive questions are not asked and it is seen that the questions do not convulse the youngsters. However, in this study, it was still ensured that the pupils' parents were aware of the interviews and gave their consent. Letters were sent to the homes of the interviewed pupils to inform their parents of the interviews. Consent forms were sent with these letter and the parents were asked to give their child their consent to participate in the interview by signing the form. Before the interview, all respondents gave their written consent to the use of the interview as research material. The participating pupils and parents received a brief introduction to the study and its objectives. The questions were designed in such a way that the objectives were approached with as familiar situations and concepts as possible. The pupils were also informed of the use of the well-being profile responses in the promotion of mental health and well-being at school. The pupils responded anonymously [36].

The study was endorsed by permissions from the Joint Municipal Authority for Primary Healthcare, the Social Welfare Board, the Board of Education, and also the Ethics Committee of the local hospital district.

### 2.5. Analysis

Grounded theory focuses on concept formation. Concepts illustrate the characteristics of the categories they contain. They express time, place, people, and people's patterns of action [32, 42].

Theoretical sensitivity is an important issue in grounded theory [37]. It means being sensitive about what data are important in developing the grounded theory. The theory has to be sensitive to the data as discovered during the research. Familiarity with relevant literature can enhance the sensitivity. In this study, the researcher also spent a lot of time discussing with the school personnel to do so. Getting familiar with the school context and with the literature also enhanced the researcher's preunderstanding about the subject [32, 38].

Both materials, the transcribed interview material and the written answers of the survey, were analysed together. First, the textual material was read several times. The analysis began by open coding. Statements the informants used to illustrate mental health promotion were picked from the text [32, 38].

Then the researcher analysed the collected material with the constant comparative method to identify similarities and differences between the statements. Each statement was compared with other statements. The aim was to classify data. The questions that were used to support the categorisation of the material included the following: “what is happening here? Who is doing something? What does this depict? What is the consequence of this?” [32, 38, 40].

The process of grouping concepts which seem to pertain to the same phenomena is called categorizing. As a result of the regrouping and comparison, all of the statements were placed in a category of statements pertaining to the same theme and a shared concept illustrating the phenomenon was identified. Each category included more than 100 statements [38, 40].  Table 1 presents an example of how the statements were placed into categories and subcategories and shows the concepts used to name the categories.

Data analysis occurred simultaneously with data collection. During the data collection categories developed in terms of their properties and dimensions. The codes produced by the analysis determined the direction of the data collection. Following the principle of saturation, the amount of data was considered sufficient when no new utterances emerged from the data collected but the same themes began to recur [32]. Sufficient sampling was determined to occur when categories offered considerable depth and breadth of understanding about the phenomenon and relationships to other categories were clear [32].

In the next phase, the axial coding, the data was examined to identify structural factors, contextual factors, and factors related to the phenomenon, circumstances, and action [32]. This way, the five key concepts which the parents and the schoolchildren used in describing the promotion of mental health in the upper-level comprehensive school emerged [32, 40].

At this stage of the research, it was important to highlight the perspective of the pupils and their families. Therefore, the analysis was left on the level of axial coding. On this level it is possible to form causal conditions, context, phenomena, and factors related to action and interaction from the material. Finally, the generated concepts were compared with the existing research information on the topic [32].

## 3. Results: Concepts of Mental Health Promotion as Described by the Schoolchildren and Their Families

With regard to mental health promotion, five key concepts were discovered in the material: “school environment,” “school friends and teachers,” “cooperation,” “actions to promote well-being and mental health,” and “getting help with problems.” Table 2 shows the key concepts related to promoting mental health and properties and describes them from the schoolchildren's and their parents' perspective.

“School environment” comprises the physical conditions and educational equipment in the school—including resources, teaching, rules, and services for pupils, such as school lunch—and their impacts on the pupils. This concept also comprises the requirements of the teaching, the flexibility and individuality of the teaching, and the characteristics of the pupils themselves.

The pupils had a lot of thoughts on the school environment. They were pleased with the high-quality teaching tools and spacious classrooms but thought that the recesses should offer more activity opportunities; just standing around is not enough. On the other hand, the rules governing recess-time activities and school practices aroused divergent opinions: some were against these rules and some wanted them to be adhered to and controlled more actively. Also, the parents considered that the school environment influences children's well-being and they had made efforts to improve the school premises, for example, via the parents' association.

*“The schoolyard could use some improving, there has been some opinions about that. About that something should be done there there (sic).”*

*“The atmosphere is good.”*

*“Needs to be improved: rules”*

Learning was considered to be a positive thing. The pupils were happy with the idea of optional subjects, but, on the other hand, they wanted to have more nontheoretical subjects such as physical exercise, music, and home economics. Also the parents wished the curricula to be more favourable for pupils who are more inclined towards artistic performance than theoretical learning. The pupils and parents wanted to add individual aspects and flexibility to the teaching and teaching methods with an increased degree of student participation. Individualism was considered to be a mental health-promoting factor in the form of promoting the self-esteem and feelings of success.

*“It's that supporting of growth and finding those positive capabilities.”*

*“The best thing in this school are (sic) the electives.”*

Learning difficulties, vandalism, and absences were further issues associated with the school environment although to a lesser extent. In general, factors related to the pupils' age and personal properties were essential in this respect. The available human and financial resources were brought up as potential obstacles to detecting and resolving problems.

*“Well, the curator-situation has been inadequate.”*

*“The school nurse is nevertheless always available every day.”*

“*School friends and teachers*” include social relationships in school and the whole school community. They have a great influence on schoolchildren's well-being. Both the children and the parents found the teacher's role very important. Friends were also considered to be important and to make the school feel like a comfortable place. Breaking up groups of friends when transferring from primary school to the upper-level comprehensive school is an issue that aroused much thought, especially in the transfer phase. Safety in the school community was also considered important.

*“I think it's that the teachers treat the pupils equally.”*

*“Nice school friends”*

The parents placed high expectations on the class teacher. The class teacher was expected to notice pupils' needs for support and respond to them. The pupils often complimented their teachers as being nice, skilled, and good teachers. However, there were also comments about too high demands and dictator-like and unfair behaviour.

*“Teachers have the expertise to see in my opinion.”*

*“I wished remedial education for my son in some of the subjects and the teacher did carry my wish forward then.”*

*“And the teachers are quite nice.”*

*Co-operation* and communication between parents and employees were things that the parents particularly expected. This concept also includes cooperation between employees and between children and adults and sufficient amount of information. The parents expected close cooperation, especially with the class teacher. The parents considered that their task is to take an interest in the child's life, monitor the child's health and motivation, and ensure sufficient rest and nutrition. Child upbringing was seen as a joint project in which parents and teachers function as partners, both relying on their own expertise; parents know the child's personality and background, whereas teachers are competent at issues related to learning.

*“I (a mother) myself try to look after resting, bedtimes.”*

* “Parents are not professionals of education.”*

According to the pupils, contacts between their homes and the school were scarce, but they believed that information will be relayed whenever necessary. The parents said that they get information on the school work from bulletins and parents' meetings. One specific channel for information is the parents' association which gathers information on the school and tries to contribute to the school environment's development by, for example, arranging fund-raising events.

*“There's not much contacting from home, but the school, however, contacts even for smaller matters.”*

*“When it's needed, everything is told. Like, when something comes up that needs to be told, the school is informed of it.”*

*“Actions to promote well-being and mental health*” are related to having an effect on enhancing the school's conditions and social environment. When talking about the factors that influence mental health, both the parents and the pupils mentioned friendship and stress. Furthermore, the parents believed that learning experiences influence adolescent's self-confidence and, consequently, mental health. The results indicated that the school had paid attention to the development of friendships and community spirit, as well as preparing for other changes related to the transition from lower-level to upper-level comprehensive school.

*“In spring, they (the new students getting to know their future school) already visited this place more than once. And then they had their future class together and they, like, already got to know each other.”*

With regard to well-being and feeling well in general, there was a lot of discussion related to the above-mentioned school environment factors, resources, and social relationships. Ensuring a sensible eating routine and a sufficient amount of sleep, improving the safety and cosiness of the school environment, fair treatment and possibilities to influence school matters were brought up as targets of action. According to the statements, these things can be influenced by the parents and the pupils, for example, via the pupils' council.

*“Well this is pretty good, you know, because there is a well arranged pupils' council in here. It has improved the cosiness.”*

The pupils said they had received some information about mental health-related issues from their teachers. Health education lessons were mentioned as one channel in learning to take care of your well-being. The parents stated that they got information about the school's practices and services in parents' meetings.

*“In parents' meetings, it's told how they do act in these situations which, for example, concern bullying.”*

“*Getting help with problems*” includes skills to notice mental health problems and possibilities to get proper and timely help. The respondents had a little knowledge of instances that offer actual mental health care services. The school health care was seen as the primary instance to contact such matters and it was considered to be easy to access. Some respondents were aware of the existence of the pupil welfare group but not of its activities or members. The respondents believed that the main responsibility for seeking help was with the individual concerned. They did not believe that the chances of detecting problems were great, especially with quiet, socially withdrawn pupils. The respondents' views on mental health work were mainly based on experienced problem situations, such as problems and negative feelings related to learning difficulties and experiences of offering help in stressful situations. Cooperation and communication between the home and the school were considered important and preferable factors with regard to mental health problems.

*“Well, we have a school nurse who's spezialised (sic) in mental health. Yeah, like, I was immediately asked whether I would like to go there to talk to him/her because I worry a lot about the exams.”*

*“Well, in my opinion, it works well now that people contact each other immediately. Like, the form teacher contacts the parents when there's something that's puzzling him or her, and the parents do the same if they are puzzled.”*

## 4. Discussion

### 4.1. Research Results in Light of Previous Research Information

In grounded theory, previous literature is used to support and to validate the findings [38]. The founded literature did not provide a comprehensive description of mental health promotion in school, but there was a good amount of studies and articles related to the individual categories. Previous studies were searched with such terms as “mental health promotion” and “school,” “children,” or “adolescents” in several databases. Several studies argued about the same kind of aspects being important as these study results did. This supports the conclusion that concepts discovered from study material are actually things that are essential in promoting mental health. “School environment” and “school friends and teachers” especially were mentioned in many studies. Barry and Jenkins [10], Konu et al. [29], Konu and Lintonen [35], and Goodman et al. [43] wrote about the importance of developing an environment that supports the well-being of the pupils. In their studies on mental health promotion projects and reviews thereof, they found strong evidence of the social competence approach, which brings focus on the promotion of resourcefulness and generic coping and competence skills.

Also “cooperation” was featured in the literature. Bickham et al. [31] see the family as a significant actor in children's services. Hopia et al. [44] found family involvement important when children were ill. Family members should help the nursing staff to gain a clearer picture of the depth and the diversity of the family health and support the resources that promote family health. Cooperation in mental health projects is also a key element in Poole's [45] project on preventive mental health work, in which families, schools, municipalities, and authorities worked in extensive cooperation to develop a model of partnership between the school and the families.

Adelman and Taylor [46, 47] named the collaborative work of school staff, health professionals, and the community resources as one very important part of successful mental health promotion work in their reviews of school mental health promotion. According to Deschesnes et al. [48] cooperation between the family, school, and community was a significant factor in the promotion of schoolchildren's mental health. In this study it was noted that the cooperation encompasses both pupils and their families. This is why it was important to include the adolescents' and families' viewpoint.

“Actions to promote mental health” in school rise from those important factors. The influence of the entire social school environment, including the teachers and their major role, is stated in the results and enhancing these elements is seen important in many studies [10, 29, 35, 43]. Barry and Jenkins [10] wrote about the significance of social and problem-solving skills for well-being. In the light of Barry's [10] results, it is easy to understand how important role the schoolchildren and their parents give to human relations and co-operation.

The results of “getting help with problems” especially reflect the viewpoints of the pupils and their families. In previous studies this aspect could not be directly seen. Anyway, previous results of co-operation [31, 44, 45, 48], mental health and social skills training [10] and being heard [49, 50] are things that also support this finding.

The importance of being heard and possibilities to have influence in one's own school matters can be seen in the results of this study. Sorsa et al. [49] wrote in their study on children's viewpoints on child psychiatric care that relationships with other children seem to be of extreme importance. According to* The UNICEF's Convention on the Rights of the Child* [50], children have a right to express their opinions and to have their views taken seriously and given due weight. Children also have a responsibility to respect the rights of others, especially those of their parents. The convention refers to the family as the fundamental group of society and the natural environment for the growth and well-being of children [50].

### 4.2. Reliability of the Research

In qualitative research, validity and reliability are not addressed in the same way as in quantitative research. Data generated using the grounded theory method can be assessed in terms of credibility, conformability, and truth value [38, 40].

The views and opinions of the parents and youngsters, on which matters are important, can be considered credible. This study features interviews with four youngsters and a survey among a large group of schoolchildren on their opinions of their school. The material from the interviews with parents was rich and versatile and described the parents' thoughts on their children's schooling from various angles. Combining several types of data proved to be a helpful way to find more information and a richer description about the studied phenomenon. For example, using the open questions from the survey supplemented the interview material remarkably.

As the material collection proceeded, certain statements became more frequent, which means that the material itself can be considered in order to confirm its own credibility. Furthermore, similar statements were found in different materials. The schoolchildren's views on mental health promotion were clearly visible [32].

The impression of the promotion of schoolchildren's mental health gained from the material provides a versatile insight into the youngsters' and parents' viewpoints from various angles. This was also the purpose of this research. A particular objective of this study was to bring out human experiences, which was well achieved with the interview material. The schoolchildren's views on mental health promotion were clearly visible [32, 40].

### 4.3. Limitations

A substantive theory produced by grounded theory can be said to be valid only for the studied population. One concern is whether the mothers' viewpoints can be extrapolated to the general family, even though they were told to represent the whole family. No fathers agreed to be interviewed, but mothers were asked to speak from the viewpoint of the whole family. At least their viewpoint can be seen to be a remarkable part of family viewpoints. However, a quality theory will inevitably identify a basic process that is also relevant more generally [40].

Details related to the school's practices and human resources vary between schools and countries, which is why detailed practices, such as group-formation on transition to the upper-level school, cannot be generalised into a description of mental health promotion practices in schools as a whole. Instead, the process of collecting and analysing the pupils' viewpoints used in this study, as well as the founded key aspects of mental health promotion in school, can be considered to apply to a wider context and internationally.

## 5. Conclusions

Grounded theory proved to be an effective mean of eliciting people's personal viewpoints. When theory is developed from the personal descriptions of the actors and the objects of action, it is easily translatable into a development tool. Pupils and their families are the key targets of mental health promotion work in schools and bringing up their viewpoints is important and forms a significant research result. The founded viewpoints help the school personnel develop their work and select the focus areas of development, even in schools with different human resources and practices [10, 26, 51].

## 6. Recommendations for Practice and Research

Schools are one of the most important settings for promoting mental health of young people. According to previous literature, the term “mental health” is often misunderstood and misused. Even professionals might only use it when discussing mental disorders. In reality, mental health is a broad concept which concerns everyone in society. Pupils and parents, as well, need knowledge of mental health promotion and problem solving in school. This study, by giving names and contents to different fields of mental health promotion, helps the whole school community to find key development areas. Communities and families should be included in the development of services. This should lead to services being better tailored to people's needs and better used.

The results indicate that the pupils find teaching and teachers to be very important factors at school. With these factors in mind, it is important to pay attention to the opportunities to increase flexibility and individuality. Finding competent teachers would also make the school work seem more worthwhile for the pupils.

Founded literature highlights the significance of theory-based preventive activities in their study. The school staff needs guidance in mental health promotion and theory-based means for the practical implementation thereof. It is often the case that the operators are not aware of the underlying theory behind development programmes, which makes the practical application more difficult. Through the perspectives of different actors, it is easier to find an understandable and practical description of the action. With the help of the produced concepts, the mental health promotion of schoolchildren can thus be observed and promoted, which is the central benefit of this study.

However, co-operation between the families and the school staff, as well as increasing the pupils' possibilities to influence, could help in this respect as well. The whole school community must be taken into consideration. Combining viewpoints of children, families, and professionals and then discussing and developing mental health promotion activities in cooperation would not only increase awareness but also enable development that takes all viewpoints into account.

The next phase of the research is to combine these different viewpoints into a substantive theory on mental health promotion in school. After this, the core category can be formed [32, 38]. The theory which will be built will help the whole school community to develop mental health promoting work by giving a framework and helping to find key development areas.

## Figures and Tables

**Table 1:** Example of statement classification.

Concept of co-operation	
Statements, phenomenon	Statement group
There's one (parents' meeting) in the autumn	General communication
Here the school makes a tuition plan, a booklet distributed to all homes	
Representatives of the police were here this autumn to talk about general safety	
Someone may also have talked about substance abuse	
We haven't had one (a parents' meeting), at least during the seventh grade	
Eight-graders have had some, like, parents' meetings	
There was one open house day when I was in some class	

And I do think that they will be in contact with the parents quite a lot	Child-specific contacts
I think it's working well now, the system that we make contact whenever something is troubling us,	
but, sure, there could be more communication between the school and home	
In my opinion it definitely needs to be more compact	
I want things to be sorted out straight away	
If there's something to tell, they will tell it (from school to home)	
If the parents haven't informed the school about missing classes in advance, we must bring an explanatory note for absence	
I personally feel that the school's contacts with my home have been very poor,	
and I use e-mail, you know	
If there's something they should know at school, they will be told.	
If I had to work out something like that, I'd make the call	

The school and home already co-operate as such partners in upbringing	Partnership in upbringing
Co-operation between the school and home is important with these things, supporting and steering and encouraging	
The teacher body gives out the message that they are here just to educate others' children	
Teachers say that parents don't care.	

**Table 2:** Key concepts and their content.

School environment	School friends and teachers	Cooperation	Actions to promote well-being and mental health	Getting help with problems
**Teaching, subjects** (i) Quality of teaching (ii) Fast pace of teaching (iii) Individuality in teaching (iv) Encouraging (v) Workload (vi) Possibilities to participate **Physical school premises/resources** (i) Cosiness and cleanliness (ii) Lunch premises (iii) Ventilation and heating **School schedules** (i) Length of school days (ii) Free periods, lunch periods, and breaks (iii) Time of starting school in the morning **Food** (i) Do pupils like the food? (ii) Significance of the lunch break and lunch itself (iii) Possibility to buy snacks and snack quality **School rules** (i) Fairness (ii) Control (iii) Safety **Resources** (i) Cooperation between authorities (ii) Human resources and different professions at school (iii) Tutoring (iv) School nurse and pupil welfare group (v) Time resources (vi) Financial resources **Pupils' situation in life** (i) Age, properties, and capabilities (ii) Transition to the upper-level comprehensive school (iii) Health (iv) Learning difficulties (v) Bullying	**Teachers** (i) Teaching skills (ii) Fairness (iii) Demands set for pupils (iv) Being nice/boring (v) Subject teachers (vi) Principal's important role **Class teacher** (i) Will he/she remain distant if he/she does not teach the class? (ii) Class teacher's classes (iii) Teachers' mental strength and time resources (iv) Considered important (v) Will they intervene in problem situations? **Friends** (i) From the neighborhood (ii) From hobbies (ii) From school (iv) Class spirit (v) Nice, good, and relaxed (vi) Class group and community (vii) Enjoying being together (viii) Can you be yourself? (ix) Safety (x) Breaking up classes when transferring to upper-level school is sad	**General cooperation between home and school** (i) Communication (ii) Open house events (iii) Parents' meetings (iv) Bulletin on school matters (v) Visitors, for example, from the police **Child-specific cooperation** (i) Discussions with class teacher (ii) Reciprocity desirable (iii) E-mail and telephone (iv) Up-to-date information (v) Things are sorted out as soon as they appear (vi) In the most serious cases the school contacts the home (vii) Could be more, free-form (viii) Contact home when there are problems (ix) Discussion face to face **Experiences of cooperation** (i) Insecurity about making the contact (ii) Difficulty to reach (iii) The school cannot contact parents with every issue (iv) Being heard/getting opinions crashed (v) Expectations do not always meet (vi) Does the contact entail questioning the other's activities? **Partnership in upbringing** (i) Mutual partnership (ii) Cooperation in supporting and encouraging (iii) Differences of opinion are sorted out (iv) Do the teachers feel that too much responsibility falls on them? **Cooperation between parents** (i) The parents' association is a channel to influence and gain information (ii) Do the parents know each other? (iii) Exchanging thoughts (iv) Activeness of parents (v) Fund-raising events	**Pupils' actions** (i) Influencing the circumstances through the pupils' union (ii) Hobbies **Parents' association actions** (i) To enhance the cosiness of premises (ii) Financial support (iii) Social skills (iv) Support for well-being (v) Support for parenthood **Teachers' actions** (i) Information about mental health; health information lessons and health education (ii) Listening and taking individuality into account (iii) Listening to pupils' suggestions (iv) Including the pupils in enhancing cosiness (v) Including the pupils in decision making (vi) Taking the school transition into account, getting to know each other, and “coaching” (vii) Information to parents (viii) Equality **Not much information on promoting mental health** (i) Especially scarce information of cases where there are no problems **Desired** Atmosphere surveys, equality	**Noticing** (i) Can you see a pupil's problems if you do not teach him/her? (ii) Disruptive behavior is easy to detect during teaching (iii) The quiet ones often go unnoticed (iv) Youngsters do not tell their parents everything (v) Home life is busy, hobbies, and so forth. (vi) It may be that no one notices the problems **Seeking and getting help** (i) The threshold to seeking help (ii) Prevent lessons (iii) Help is available (iv) Referral to further instances (v) The school nurse is easy to approach (vi) The pupil's own initiative (vii) Early intervention is important (viii) Teacher notices when teaching is disturbed (ix) Pupil seeks help him/herself (x) Goes to talk (xi) Help is offered (xii) Slow intervention **Experiences** (i) Examination of symptoms was not available in time (ii) Parents demand (iii) Joint meetings **Improvement suggestions** (i) Addressing bullying (ii) Help should be offered automatically in risk cases
